# Crystal structure of (*E*)-1-(2,4-di­nitro­phen­yl)-2-[(*E*)-5-phenyl-1-(*p*-tol­yl)pent-2-en-4-yn-1-yl­idene]hydrazine

**DOI:** 10.1107/S2056989015019301

**Published:** 2015-10-17

**Authors:** Alexander A. Golovanov, Anna V. Vologzhanina, Evgeniya D. Voronova, Vadim V. Bekin, Sergey V. Naumov

**Affiliations:** a Department of Chemistry Chemical Processes and Technologies, Togliatti State University, 445667 Togliatti, Russian Federation; b Nesmeyanov Institute of Organoelement Compounds of Russian Academy of Sciences, 119991 Moscow, Russian Federation; c Laboratory of Functional of Heterocyclic Compounds, Togliatti State University, 445667 Togliatti, Russian Federation

**Keywords:** crystal structure, hydrazones, hydrazine, hydrogen bonding, C—H⋯π inter­actions, π–π inter­actions

## Abstract

In the title compound, C_24_H_18_N_4_O_4_, the plane of the phenyl ring is inclined to those of the toluene ring and the di­nitro-substituted benzene ring by 66.96 (19) and 47.06 (18)°, respectively, while the planes of the two benzene rings are inclined to one another by 36.26 (19)°. There is an intra­molecular N—H⋯O hydrogen bond between the NH group and the O atom of a nitro group, forming an *S*(6) ring motif. In the crystal, mol­ecules are linked by C—H⋯O hydrogen bonds and C—H⋯π inter­actions, forming a three-dimensional network. There are also weak π–π inter­actions present involving the phenyl ring and the di­nitro-substituted benzene ring [inter-centroid distance = 3.741 (2) Å].

## Related literature   

For the biological activity of chalcones, and their aryl­thio-containing derivatives, see: Nielsen *et al.* (2005 ▸); Wu *et al.* (2011 ▸); Chate *et al.* (2012 ▸); Karaman *et al.* (2012 ▸). For the synthesis and crystal structure of 1,5-di­aryl­pent-2-en-4-yn-1-one precursors, see: Golovanov *et al.* (2013 ▸); Vologzhanina *et al.* (2014 ▸).
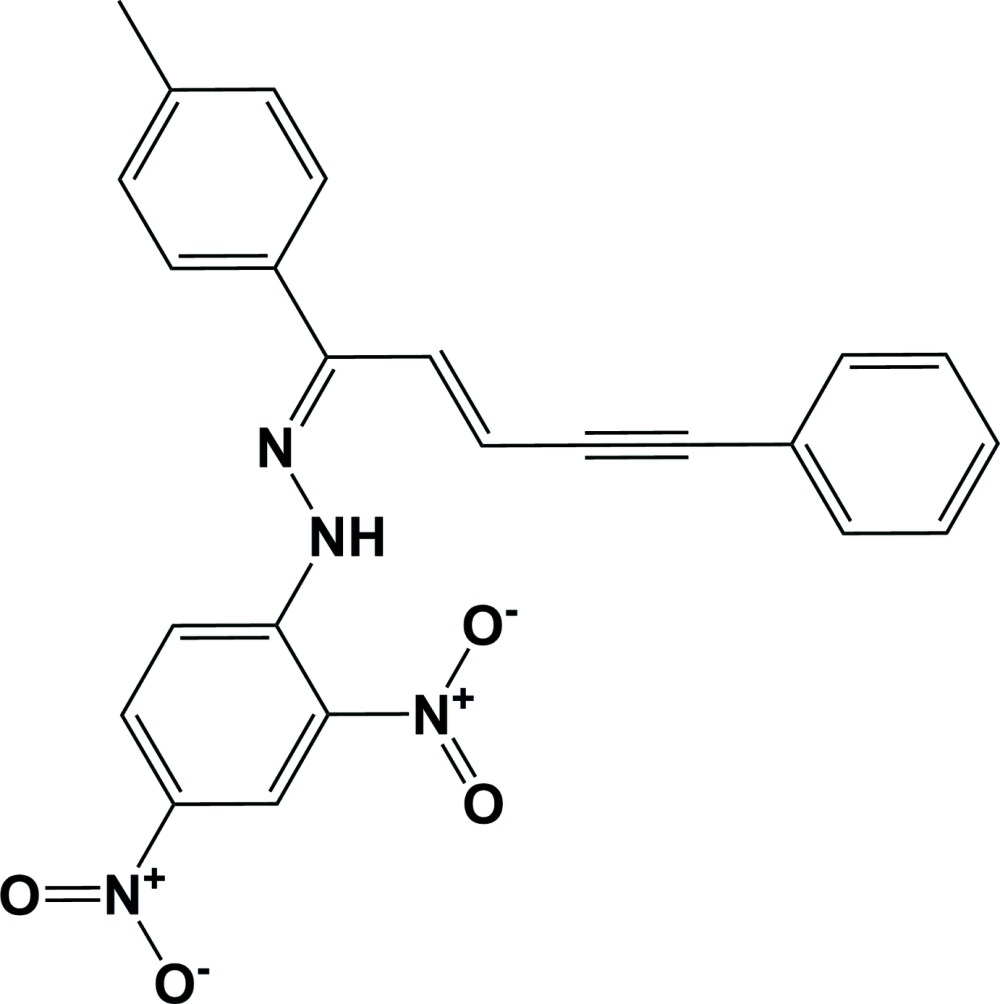


## Experimental   

### Crystal data  

C_24_H_18_N_4_O_4_
*M* *_r_* = 426.42
Monoclinic, 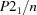

*a* = 18.4810 (6) Å
*b* = 6.1674 (2) Å
*c* = 19.2366 (12) Å
β = 109.902 (5)°
*V* = 2061.63 (17) Å^3^
*Z* = 4
Cu *K*α radiation
μ = 0.79 mm^−1^
*T* = 120 K
0.42 × 0.06 × 0.06 mm

### Data collection  

Bruker APEXII CCD diffractometer
Absorption correction: multi-scan (*SADABS*; Bruker, 2005 ▸) *T*_min_ = 0.903, *T*_max_ = 0.916
27966 measured reflections
3661 independent reflections
2388 reflections with *I* > 2σ(*I*)
*R*_int_ = 0.174

### Refinement  

*R*[*F* ^2^ > 2σ(*F* ^2^)] = 0.091
*wR*(*F* ^2^) = 0.283
*S* = 1.03
3661 reflections
295 parameters
H atoms treated by a mixture of independent and constrained refinement
Δρ_max_ = 0.53 e Å^−3^
Δρ_min_ = −0.49 e Å^−3^

### 

Data collection: *APEX2* (Bruker, 2005 ▸); cell refinement: *SAINT* (Bruker, 2005 ▸); data reduction: *SAINT*; program(s) used to solve structure: *SHELXS97* (Sheldrick, 2008 ▸); program(s) used to refine structure: *SHELXL2014* (Sheldrick, 2015 ▸); molecular graphics: *OLEX2* (Dolomanov *et al.*, 2009 ▸); software used to prepare material for publication: *OLEX2*.

## Supplementary Material

Crystal structure: contains datablock(s) Global, I. DOI: 10.1107/S2056989015019301/su5223sup1.cif

Structure factors: contains datablock(s) I. DOI: 10.1107/S2056989015019301/su5223Isup2.hkl

Supporting information file. DOI: 10.1107/S2056989015019301/su5223Isup3.cml

**Fig. 1.**
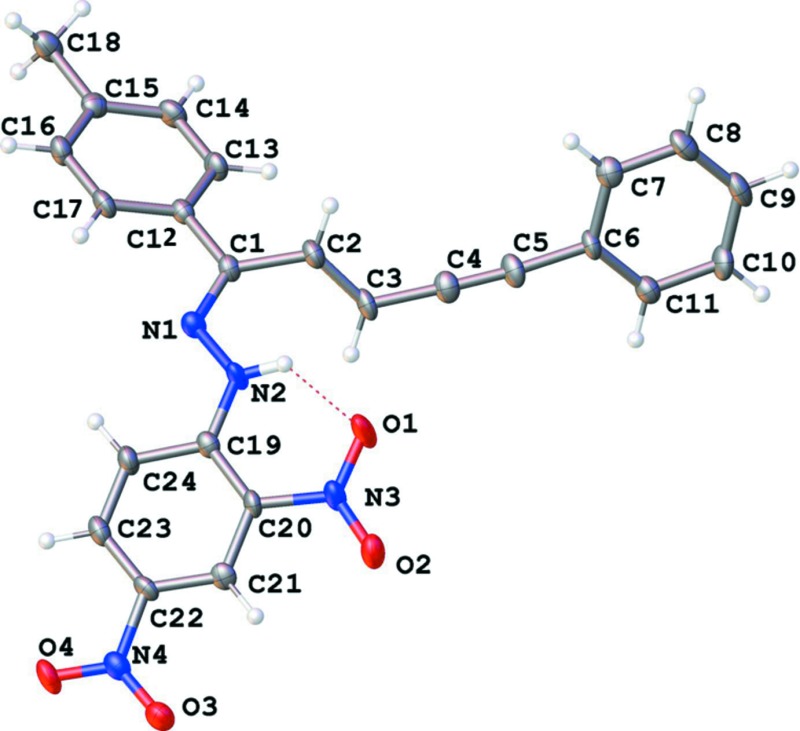
The molecular structure of the title compound, with atom labelling. Displacement ellipsoids are drawn at the 50% probability level.

**Fig. 2.**
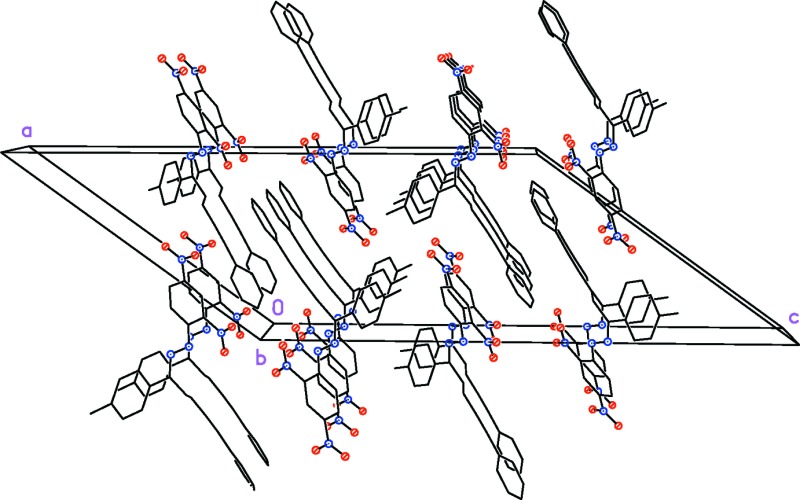
A partial view along the b axis of the crystal packing of the title compound. The H atoms have been omitted.

. DOI: 10.1107/S2056989015019301/su5223fig1.tif
The mol­ecular structure of the title compound, with atom labelling. Displacement ellipsoids are drawn at the 50% probability level.

b . DOI: 10.1107/S2056989015019301/su5223fig2.tif
A partial view along the *b* axis of the crystal packing of the title compound. The H atoms have been omitted.

CCDC reference: 1430774

Additional supporting information:  crystallographic information; 3D view; checkCIF report

## Figures and Tables

**Table 1:** Hydrogen-bond geometry (, ) *Cg*2 is the centroid of the C12C17 ring.

*D*H*A*	*D*H	H*A*	*D* *A*	*D*H*A*
N2H2*N*O1	0.89(5)	1.86(5)	2.597(5)	139(4)
C8H8O1^i^	0.95	2.49	3.396(5)	160
C10H10O2^ii^	0.95	2.51	3.337(5)	146
C3H3*Cg*2^iii^	0.95	2.63	3.504(4)	153

Symmetry codes: (i) 
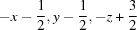
; (ii) 
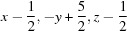
; (iii) 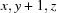.

## supplementary crystallographic information

### S1. Comments

Chalcones exhibit anti­biotic (Nielsen *et al.*, 2005) and anti-inflammatory (Wu *et al.*, 2011) activity. Aryl­thio-containing ketones are also active against some human pathogenic microorganisms (Chate *et al.*, 2012; Karaman *et al.*, 2012). Thus, a molecule which contains both fragments may have a high biological effect. Herein we present the synthesis and crystal structure of the title hydrazone, prepared by the reaction between 2,4-di­nitro­phenyl­hydrazine and 1-(4-methyl­phenyl)-5-phenyl-2-penten-4-yn-1-one.

In the title compound, Fig. 1, the length of the C3—C4 bond [1.427 (6) Å] indicates slight delocalization of electron density along the polyene C═ C—C═C chain. In contrast with the parent pent-2-en-4-yn-1-one compound (Vologzhanina *et al.*, 2014) the benzene rings at C1 and C5 atoms are twisted with respect to one another, with a dihedral angle of 66.96 (19) °. There is an intra­molecular N—H···O hydrogen bond, between an O atom of a nitro-group and the NH H atom forming an S(6) ring motif (Table 1 and Fig. 1).

In the crystal, molecules are linked by C—H···O hydrogen bonds and C—H···π inter­actions, forming a three-dimensional structure (Table 1 and Fig. 2). There are also weak π-π inter­actions present involving the phenyl ring and the di­nitro-substituted benzene ring [Cg1···Cg3^i^ = 3.741 (2) Å; Cg1 and Cg3 are the centroids of rings C6—C11 and C19—C24, respectively; symmetry code: x-1/2, -y+3/2, z-1/2].

### S2. Synthesis and crystallization

A mixture of 2,4-di­nitro­phenyl­hydrazine (320 mg, 1.61 mmol), 1-(4-methyl­phenyl)-5-phenyl-2-penten-4-yn-1-one (374 mg, 1.61 mmol) and 1 ml concentrated HCl were dissolved in MeOH (20 ml). The reaction mixture was heated under reflux. The mixture was cooled, and the precipitate of the hydro­zone was filtered off, washed on a filter with 2 ml of cold 95% EtOH, and dried (yield 87%). The single-crystals of the title compound were obtained by slow crystallization of a solution in MeOH (m.p.: 415–417 K). IR (KBr), ν/cm^-1^: 2196, 1615. ^1^H NMR (400 MHz, CDCl_3_): 2.44 (s, 3H), 6.43 (d, 1H, J = 16.3 Hz), 7.07 (d, 1H, J = 16.3 Hz), 7.32 (m, 5H), 7.53 (dd, 2H, J = 4.3 Hz, J = 3.0 Hz), 7.61 (d, 2H, J =8.0 Hz), 8.10 (d, 1H, J = 9.0 Hz), 8.35 (dd, 1H, J = 9.6 Hz, J = 2.0 Hz), 9.17 (d, 1H, J = 2.3 Hz), 11.65 (s, 1H). Anal. Calcd. for C_24_H_18_N_4_O_4_: C, 67.60; H, 4.26. Found: C, 67.95; H, 4.08.

### S3. Refinement

Crystal data, data collection and structure refinement details are summarized in Table 2. The NH H atom was located in a difference Fourier map and freely refined. The C-bound H atoms were included in calculated positions and treated as riding atoms: C—H = 0.95-0.98 Å with *U*_iso_(H) = 1.5*U*_eq_(C-methyl) and 1.2U_eq_(C) for other H atoms.

### Crystal data

**Table:**

C_24_H_18_N_4_O_4_	*F*(000) = 888
*M**_r_* = 426.42	*D*_x_ = 1.374 Mg m^−^^3^
Monoclinic, *P*2_1_/*n*	Cu *K*α radiation, λ = 1.54178 Å
*a* = 18.4810 (6) Å	Cell parameters from 4900 reflections
*b* = 6.1674 (2) Å	θ = 2.9–66.5°
*c* = 19.2366 (12) Å	µ = 0.79 mm^−^^1^
β = 109.902 (5)°	*T* = 120 K
*V* = 2061.63 (17) Å^3^	Needle, red
*Z* = 4	0.42 × 0.06 × 0.06 mm

### Data collection

**Table:**

Bruker APEXII CCD diffractometer	3661 independent reflections
Radiation source: fine-focus sealed tube	2388 reflections with *I* > 2σ(*I*)
Graphite monochromator	*R*_int_ = 0.174
φ and ω scans	θ_max_ = 68.1°, θ_min_ = 2.9°
Absorption correction: multi-scan (*SADABS*; Bruker, 2005)	*h* = −22→22
*T*_min_ = 0.903, *T*_max_ = 0.916	*k* = −7→7
27966 measured reflections	*l* = −21→22

### Refinement

**Table:**

Refinement on *F*^2^	Secondary atom site location: difference Fourier map
Least-squares matrix: full	Hydrogen site location: mixed
*R*[*F*^2^ > 2σ(*F*^2^)] = 0.091	H atoms treated by a mixture of independent and constrained refinement
*wR*(*F*^2^) = 0.283	*w* = 1/[σ^2^(*F*_o_^2^) + (0.1877*P*)^2^ + 0.4158*P*] where *P* = (*F*_o_^2^ + 2*F*_c_^2^)/3
*S* = 1.03	(Δ/σ)_max_ < 0.001
3661 reflections	Δρ_max_ = 0.53 e Å^−^^3^
295 parameters	Δρ_min_ = −0.49 e Å^−^^3^
0 restraints	Extinction correction: *SHELXL2014* (Sheldrick, 2015), Fc^*^=kFc[1+0.001xFc^2^λ^3^/sin(2θ)]^-1/4^
Primary atom site location: structure-invariant direct methods	Extinction coefficient: 0.0043 (10)

### Special details

**Table:**

Geometry. All esds (except the esd in the dihedral angle between two l.s. planes) are estimated using the full covariance matrix. The cell esds are taken into account individually in the estimation of esds in distances, angles and torsion angles; correlations between esds in cell parameters are only used when they are defined by crystal symmetry. An approximate (isotropic) treatment of cell esds is used for estimating esds involving l.s. planes.

### Fractional atomic coordinates and isotropic or equivalent isotropic displacement parameters (Å^2^)

**Table:**

	*x*	*y*	*z*	*U*_iso_*/*U*_eq_	
O1	0.03666 (16)	0.8320 (6)	0.91286 (19)	0.0415 (10)	
O2	0.08785 (16)	1.1218 (6)	0.97070 (17)	0.0347 (9)	
O3	0.35551 (17)	1.2102 (5)	1.08437 (18)	0.0343 (8)	
O4	0.43421 (15)	0.9726 (5)	1.06733 (17)	0.0337 (9)	
N1	0.13617 (18)	0.3687 (6)	0.84326 (18)	0.0235 (9)	
N2	0.12194 (18)	0.5366 (6)	0.88310 (19)	0.0239 (9)	
H2N	0.077 (3)	0.598 (8)	0.877 (3)	0.036 (14)*	
N3	0.09357 (18)	0.9408 (6)	0.94809 (19)	0.0253 (9)	
N4	0.36865 (19)	1.0372 (6)	1.05984 (19)	0.0256 (9)	
C1	0.0782 (2)	0.2979 (7)	0.7893 (2)	0.0218 (10)	
C2	−0.0018 (2)	0.3818 (7)	0.7662 (2)	0.0237 (10)	
H2	−0.0418	0.2810	0.7626	0.028*	
C3	−0.0214 (2)	0.5874 (7)	0.7502 (2)	0.0239 (10)	
H3	0.0183	0.6913	0.7564	0.029*	
C4	−0.0994 (2)	0.6596 (8)	0.7240 (2)	0.0267 (10)	
C5	−0.1651 (2)	0.7169 (8)	0.7004 (2)	0.0261 (10)	
C6	−0.2456 (2)	0.7682 (7)	0.6685 (2)	0.0229 (10)	
C7	−0.3002 (2)	0.6081 (8)	0.6657 (2)	0.0278 (11)	
H7	−0.2841	0.4694	0.6868	0.033*	
C8	−0.3780 (2)	0.6527 (8)	0.6322 (2)	0.0307 (11)	
H8	−0.4150	0.5436	0.6301	0.037*	
C9	−0.4020 (2)	0.8530 (8)	0.6020 (2)	0.0293 (11)	
H9	−0.4553	0.8816	0.5785	0.035*	
C10	−0.3485 (2)	1.0128 (8)	0.6058 (2)	0.0292 (11)	
H10	−0.3650	1.1522	0.5857	0.035*	
C11	−0.2702 (2)	0.9698 (7)	0.6390 (2)	0.0268 (10)	
H11	−0.2336	1.0802	0.6414	0.032*	
C12	0.0927 (2)	0.1107 (7)	0.7471 (2)	0.0210 (9)	
C13	0.0446 (2)	0.0721 (7)	0.6754 (2)	0.0260 (10)	
H13	0.0018	0.1644	0.6534	0.031*	
C14	0.0585 (2)	−0.1003 (7)	0.6353 (2)	0.0275 (11)	
H14	0.0253	−0.1240	0.5859	0.033*	
C15	0.1208 (2)	−0.2392 (7)	0.6668 (2)	0.0245 (10)	
C16	0.1688 (2)	−0.2011 (7)	0.7387 (2)	0.0261 (10)	
H16	0.2108	−0.2960	0.7611	0.031*	
C17	0.1563 (2)	−0.0273 (7)	0.7783 (2)	0.0251 (10)	
H17	0.1908	−0.0003	0.8269	0.030*	
C18	0.1338 (2)	−0.4305 (8)	0.6238 (3)	0.0331 (12)	
H18C	0.1508	−0.5552	0.6569	0.050*	
H18B	0.1733	−0.3941	0.6023	0.050*	
H18A	0.0856	−0.4667	0.5841	0.050*	
C19	0.1805 (2)	0.6534 (7)	0.9298 (2)	0.0218 (10)	
C20	0.1700 (2)	0.8485 (7)	0.9621 (2)	0.0207 (9)	
C21	0.2312 (2)	0.9736 (7)	1.0058 (2)	0.0216 (10)	
H21	0.2226	1.1075	1.0261	0.026*	
C22	0.3048 (2)	0.8976 (7)	1.0187 (2)	0.0221 (10)	
C23	0.3174 (2)	0.7040 (8)	0.9893 (2)	0.0251 (10)	
H23	0.3687	0.6549	0.9996	0.030*	
C24	0.2583 (2)	0.5810 (7)	0.9458 (2)	0.0261 (10)	
H24	0.2685	0.4476	0.9261	0.031*	

### Atomic displacement parameters (Å^2^)

**Table:**

	*U*^11^	*U*^22^	*U*^33^	*U*^12^	*U*^13^	*U*^23^
O1	0.0141 (15)	0.054 (2)	0.049 (2)	0.0019 (15)	0.0020 (14)	−0.0209 (18)
O2	0.0210 (15)	0.045 (2)	0.0337 (19)	0.0090 (14)	0.0032 (13)	−0.0054 (16)
O3	0.0243 (16)	0.036 (2)	0.0363 (19)	−0.0025 (14)	0.0024 (13)	−0.0077 (15)
O4	0.0146 (14)	0.048 (2)	0.0335 (18)	0.0014 (14)	0.0018 (12)	−0.0048 (15)
N1	0.0194 (17)	0.030 (2)	0.0198 (18)	0.0003 (15)	0.0045 (14)	−0.0016 (15)
N2	0.0113 (16)	0.036 (2)	0.0210 (19)	0.0008 (15)	0.0010 (14)	−0.0081 (16)
N3	0.0165 (17)	0.034 (2)	0.0212 (19)	0.0048 (16)	0.0011 (14)	−0.0079 (16)
N4	0.0204 (18)	0.033 (2)	0.0205 (19)	−0.0014 (16)	0.0033 (14)	0.0011 (16)
C1	0.0145 (19)	0.031 (3)	0.019 (2)	0.0018 (17)	0.0055 (16)	0.0040 (17)
C2	0.0153 (19)	0.032 (3)	0.023 (2)	0.0005 (18)	0.0053 (16)	0.0000 (19)
C3	0.0149 (19)	0.036 (3)	0.017 (2)	0.0038 (18)	0.0001 (15)	0.0029 (18)
C4	0.022 (2)	0.036 (3)	0.020 (2)	0.0042 (19)	0.0058 (17)	0.0005 (19)
C5	0.021 (2)	0.037 (3)	0.016 (2)	0.0047 (19)	−0.0004 (16)	0.0026 (18)
C6	0.0140 (18)	0.033 (3)	0.019 (2)	0.0037 (17)	0.0017 (15)	0.0028 (18)
C7	0.027 (2)	0.036 (3)	0.019 (2)	0.001 (2)	0.0067 (18)	0.0023 (19)
C8	0.019 (2)	0.045 (3)	0.029 (2)	−0.005 (2)	0.0081 (18)	−0.002 (2)
C9	0.016 (2)	0.046 (3)	0.024 (2)	0.001 (2)	0.0044 (17)	−0.004 (2)
C10	0.022 (2)	0.039 (3)	0.023 (2)	0.010 (2)	0.0021 (17)	0.007 (2)
C11	0.021 (2)	0.031 (3)	0.025 (2)	0.0005 (19)	0.0038 (17)	0.0026 (18)
C12	0.0152 (18)	0.030 (3)	0.017 (2)	0.0005 (17)	0.0045 (15)	0.0024 (17)
C13	0.019 (2)	0.032 (3)	0.021 (2)	0.0030 (18)	−0.0013 (17)	0.0017 (19)
C14	0.022 (2)	0.035 (3)	0.020 (2)	−0.0017 (19)	0.0011 (17)	−0.0041 (19)
C15	0.021 (2)	0.027 (3)	0.027 (2)	−0.0035 (18)	0.0115 (17)	−0.0019 (18)
C16	0.0173 (19)	0.033 (3)	0.025 (2)	0.0044 (18)	0.0032 (17)	0.0001 (19)
C17	0.0133 (19)	0.037 (3)	0.021 (2)	−0.0007 (18)	0.0008 (16)	0.0014 (19)
C18	0.023 (2)	0.045 (3)	0.031 (3)	−0.001 (2)	0.0092 (19)	−0.010 (2)
C19	0.017 (2)	0.031 (3)	0.016 (2)	0.0003 (17)	0.0042 (16)	0.0017 (17)
C20	0.0126 (19)	0.028 (3)	0.018 (2)	0.0026 (16)	0.0005 (15)	0.0028 (17)
C21	0.023 (2)	0.029 (3)	0.0111 (19)	0.0003 (18)	0.0024 (15)	0.0005 (17)
C22	0.0154 (19)	0.033 (3)	0.014 (2)	−0.0037 (18)	0.0002 (15)	0.0028 (17)
C23	0.0135 (19)	0.037 (3)	0.023 (2)	0.0015 (18)	0.0034 (16)	0.0007 (19)
C24	0.017 (2)	0.033 (3)	0.025 (2)	0.0033 (18)	0.0033 (17)	0.0020 (19)

### Geometric parameters (Å, º)

**Table:**

O1—N3	1.237 (4)	C10—C11	1.394 (6)
O2—N3	1.215 (4)	C10—H10	0.9500
O3—N4	1.224 (5)	C11—H11	0.9500
O4—N4	1.236 (4)	C12—C13	1.383 (6)
N1—C1	1.288 (5)	C12—C17	1.409 (6)
N1—N2	1.366 (5)	C13—C14	1.388 (6)
N2—C19	1.355 (5)	C13—H13	0.9500
N2—H2N	0.89 (5)	C14—C15	1.396 (6)
N3—C20	1.461 (5)	C14—H14	0.9500
N4—C22	1.457 (5)	C15—C16	1.387 (6)
C1—C2	1.484 (5)	C15—C18	1.507 (6)
C1—C12	1.488 (6)	C16—C17	1.378 (6)
C2—C3	1.326 (6)	C16—H16	0.9500
C2—H2	0.9500	C17—H17	0.9500
C3—C4	1.427 (6)	C18—H18C	0.9800
C3—H3	0.9500	C18—H18B	0.9800
C4—C5	1.195 (6)	C18—H18A	0.9800
C5—C6	1.439 (5)	C19—C20	1.398 (6)
C6—C11	1.377 (6)	C19—C24	1.435 (5)
C6—C7	1.400 (6)	C20—C21	1.391 (6)
C7—C8	1.388 (6)	C21—C22	1.379 (6)
C7—H7	0.9500	C21—H21	0.9500
C8—C9	1.373 (7)	C22—C23	1.374 (6)
C8—H8	0.9500	C23—C24	1.358 (6)
C9—C10	1.380 (6)	C23—H23	0.9500
C9—H9	0.9500	C24—H24	0.9500
			
C1—N1—N2	116.2 (3)	C17—C12—C1	121.0 (4)
C19—N2—N1	120.8 (3)	C12—C13—C14	120.6 (4)
C19—N2—H2N	112 (3)	C12—C13—H13	119.7
N1—N2—H2N	127 (3)	C14—C13—H13	119.7
O2—N3—O1	122.2 (3)	C13—C14—C15	120.6 (4)
O2—N3—C20	119.2 (3)	C13—C14—H14	119.7
O1—N3—C20	118.6 (4)	C15—C14—H14	119.7
O3—N4—O4	123.4 (4)	C16—C15—C14	118.8 (4)
O3—N4—C22	119.6 (3)	C16—C15—C18	120.9 (4)
O4—N4—C22	116.9 (4)	C14—C15—C18	120.3 (4)
N1—C1—C2	126.5 (4)	C17—C16—C15	120.8 (4)
N1—C1—C12	116.6 (3)	C17—C16—H16	119.6
C2—C1—C12	117.0 (3)	C15—C16—H16	119.6
C3—C2—C1	124.6 (4)	C16—C17—C12	120.4 (4)
C3—C2—H2	117.7	C16—C17—H17	119.8
C1—C2—H2	117.7	C12—C17—H17	119.8
C2—C3—C4	122.9 (4)	C15—C18—H18C	109.5
C2—C3—H3	118.5	C15—C18—H18B	109.5
C4—C3—H3	118.5	H18C—C18—H18B	109.5
C5—C4—C3	178.3 (5)	C15—C18—H18A	109.5
C4—C5—C6	174.9 (5)	H18C—C18—H18A	109.5
C11—C6—C7	119.2 (4)	H18B—C18—H18A	109.5
C11—C6—C5	121.5 (4)	N2—C19—C20	123.5 (4)
C7—C6—C5	119.3 (4)	N2—C19—C24	119.6 (4)
C8—C7—C6	119.8 (4)	C20—C19—C24	116.9 (4)
C8—C7—H7	120.1	C21—C20—C19	122.6 (4)
C6—C7—H7	120.1	C21—C20—N3	115.6 (4)
C9—C8—C7	120.6 (4)	C19—C20—N3	121.7 (3)
C9—C8—H8	119.7	C22—C21—C20	118.0 (4)
C7—C8—H8	119.7	C22—C21—H21	121.0
C8—C9—C10	119.9 (4)	C20—C21—H21	121.0
C8—C9—H9	120.1	C23—C22—C21	121.0 (4)
C10—C9—H9	120.1	C23—C22—N4	121.2 (4)
C9—C10—C11	120.1 (4)	C21—C22—N4	117.6 (4)
C9—C10—H10	120.0	C24—C23—C22	121.7 (4)
C11—C10—H10	120.0	C24—C23—H23	119.1
C6—C11—C10	120.4 (4)	C22—C23—H23	119.1
C6—C11—H11	119.8	C23—C24—C19	119.6 (4)
C10—C11—H11	119.8	C23—C24—H24	120.2
C13—C12—C17	118.7 (4)	C19—C24—H24	120.2
C13—C12—C1	120.2 (4)		
			
C1—N1—N2—C19	−165.5 (4)	C13—C12—C17—C16	−2.0 (6)
N2—N1—C1—C2	0.5 (6)	C1—C12—C17—C16	179.5 (4)
N2—N1—C1—C12	−178.2 (3)	N1—N2—C19—C20	168.5 (4)
N1—C1—C2—C3	54.1 (6)	N1—N2—C19—C24	−9.2 (6)
C12—C1—C2—C3	−127.2 (5)	N2—C19—C20—C21	−175.4 (4)
C1—C2—C3—C4	176.3 (4)	C24—C19—C20—C21	2.4 (6)
C11—C6—C7—C8	1.3 (6)	N2—C19—C20—N3	0.5 (6)
C5—C6—C7—C8	−177.2 (4)	C24—C19—C20—N3	178.3 (4)
C6—C7—C8—C9	−0.4 (7)	O2—N3—C20—C21	4.4 (6)
C7—C8—C9—C10	−0.9 (7)	O1—N3—C20—C21	−176.3 (4)
C8—C9—C10—C11	1.1 (7)	O2—N3—C20—C19	−171.9 (4)
C7—C6—C11—C10	−1.1 (6)	O1—N3—C20—C19	7.5 (6)
C5—C6—C11—C10	177.4 (4)	C19—C20—C21—C22	−1.6 (6)
C9—C10—C11—C6	−0.1 (7)	N3—C20—C21—C22	−177.8 (3)
N1—C1—C12—C13	−157.2 (4)	C20—C21—C22—C23	0.0 (6)
C2—C1—C12—C13	24.0 (6)	C20—C21—C22—N4	175.5 (3)
N1—C1—C12—C17	21.3 (6)	O3—N4—C22—C23	178.9 (4)
C2—C1—C12—C17	−157.5 (4)	O4—N4—C22—C23	−0.4 (6)
C17—C12—C13—C14	0.5 (6)	O3—N4—C22—C21	3.4 (6)
C1—C12—C13—C14	179.0 (4)	O4—N4—C22—C21	−175.9 (4)
C12—C13—C14—C15	0.7 (7)	C21—C22—C23—C24	0.8 (7)
C13—C14—C15—C16	−0.4 (6)	N4—C22—C23—C24	−174.6 (4)
C13—C14—C15—C18	177.8 (4)	C22—C23—C24—C19	0.0 (7)
C14—C15—C16—C17	−1.1 (6)	N2—C19—C24—C23	176.4 (4)
C18—C15—C16—C17	−179.3 (4)	C20—C19—C24—C23	−1.5 (6)
C15—C16—C17—C12	2.3 (7)		

### Hydrogen-bond geometry (Å, º)

Cg2 is the centroid of the C12–C17 ring.

**Table:**

*D*—H···*A*	*D*—H	H···*A*	*D*···*A*	*D*—H···*A*
N2—H2*N*···O1	0.89 (5)	1.86 (5)	2.597 (5)	139 (4)
C8—H8···O1^i^	0.95	2.49	3.396 (5)	160
C10—H10···O2^ii^	0.95	2.51	3.337 (5)	146
C3—H3···*Cg*2^iii^	0.95	2.63	3.504 (4)	153

Symmetry codes: (i) −*x*−1/2, *y*−1/2, −*z*+3/2; (ii) *x*−1/2, −*y*+5/2, *z*−1/2; (iii) *x*, *y*+1, *z*.
