# Supplementary figures and images for: Crystal structure of (E)-1-(2,4-di­nitro­phen­yl)-2-[(E)-5-phenyl-1-(p-tol­yl)pent-2-en-4-yn-1-yl­idene]hydrazine
Source: Acta Crystallogr E Crystallogr Commun. 2015 Oct 17;71(Pt 11):o846–7. doi: 10.1107/S2056989015019301 (PMC4645032; doi:10.1107/S2056989015019301)

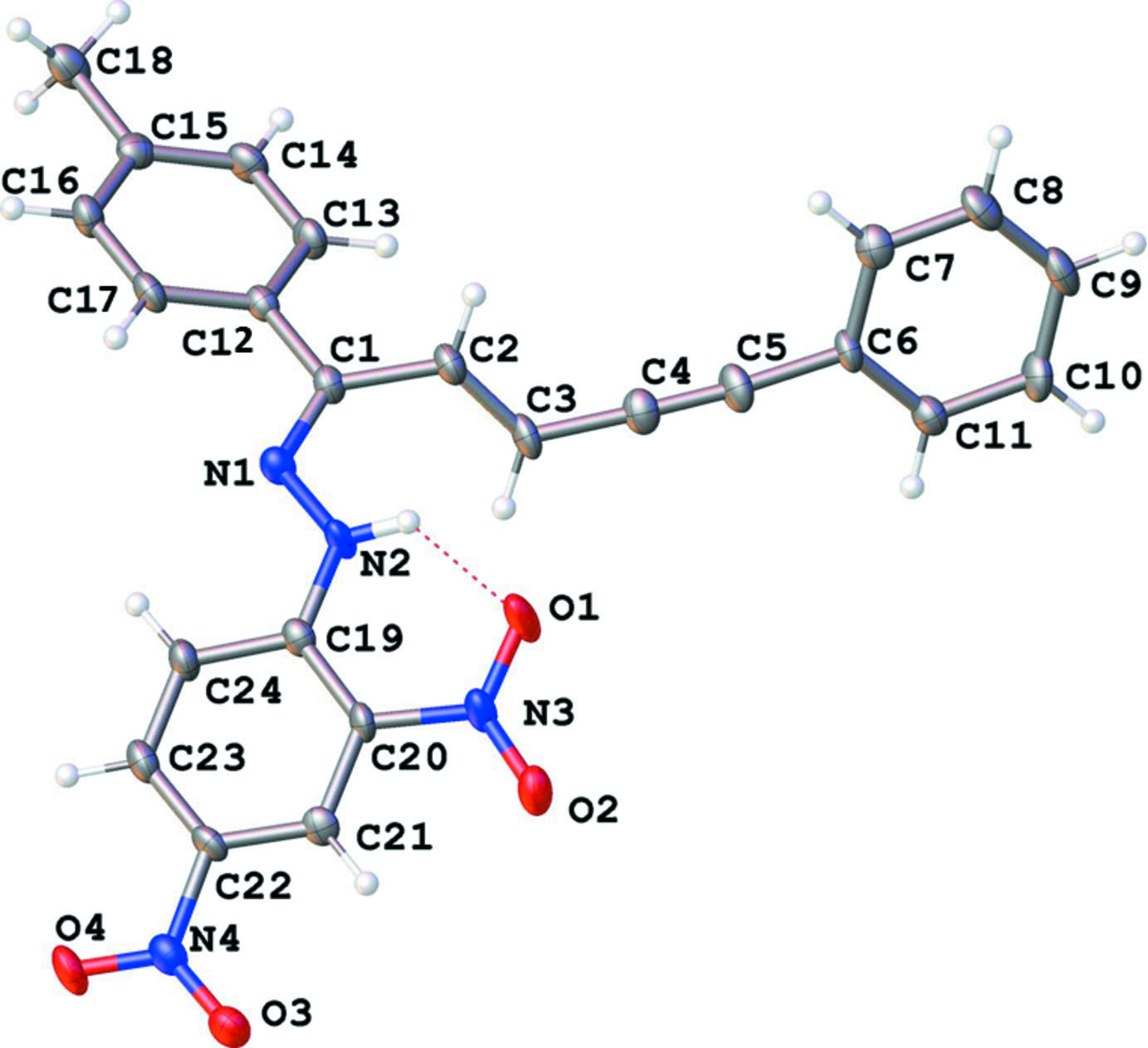

Supplement: Supplementary file 4 [file e-71-0o846-fig1.tif]

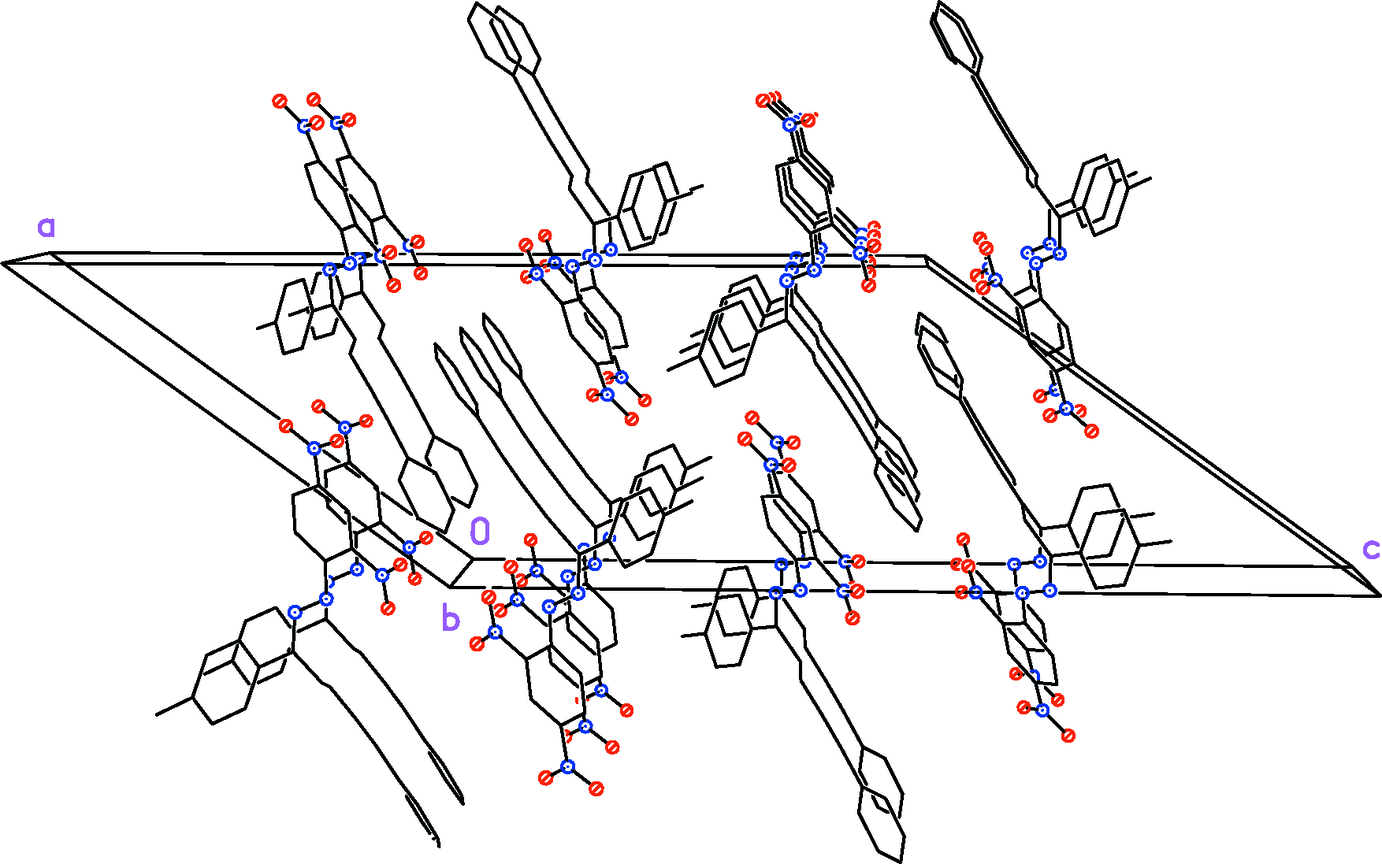

Supplement: Supplementary file 5 [file e-71-0o846-fig2.tif]
